# Cancer-associated adipocytes: key players in breast cancer progression

**DOI:** 10.1186/s13045-019-0778-6

**Published:** 2019-09-10

**Authors:** Qi Wu, Bei Li, Zhiyu Li, Juanjuan Li, Si Sun, Shengrong Sun

**Affiliations:** 1 Department of Breast and Thyroid Surgery, Renmin Hospital of Wuhan University, 238 Ziyang Road, Wuhan, Hubei People’s Republic of China; 2 Metabolomics and Cell Biology Platforms, Gustave Roussy Comprehensive Cancer Institute, Villejuif, France; 3 Faculty of Medicine, University of Paris Sud-Saclay, Kremlin-Bicêtre, France; 4 Department of Clinical Laboratory, Renmin Hospital of Wuhan University, 238 Ziyang Road, Wuhan, Hubei People’s Republic of China

**Keywords:** Breast cancer, cancer-associated adipocyte, exosome, miRNAs

## Abstract

Adipocytes are one of the primary stromal cells in many tissues, and they are considered to play an active role in the tumor microenvironment. Cancer-associated adipocytes (CAAs) are not only found adjacent to cancer cells, but also communicate with cancer cells through releasing various factors that can mediate local and systemic effects. The adipocyte-cancer cell crosstalk leads to phenotypical and functional changes of both cell types, which can further enhance tumor progression. Indeed, obesity, which is associated with an increase in adipose mass and an alteration of adipose tissue, is becoming pandemic in some countries and it is now considered to be an independent risk factor for cancer progression. In this review, we focus on the potential mechanisms involved with special attention to the adipocyte-cancer cell circle in breast cancer. We envisage that besides having a direct impact on tumor cells, CAAs systemically preconditions the tumor microenvironment by favoring anti-tumor immunity. A better understanding of cancer-associated adipocytes and the key molecular events in the adipocyte-cancer cell crosstalk will provide insights into tumor biology and permit the optimization of therapeutic strategies.

## Introduction

The tumor microenvironment (TME) is a heterogeneous ecosystem composed of infiltrating immune cells, mesenchymal support cells, and matrix components contributing to tumor progression. Adipocytes are the primary cellular components comprising the breast cancer (BC) microenvironment, and emerging evidence indicates that adipocytes drive enhanced tumor progression through mutual and dynamic communication between tumor cells and adipocytes [1, 2]. Specifically, normal adipocytes are driven into cancer-associated adipocytes (CAAs) by tumor cells and these tumor cells become metabolic parasites, which are identified by their seizing of metabolites such as ketone bodies, fatty acids, pyruvate, and lactate from stromal adipocytes [3–5]. This review will summarize the importance of CAAs in the biological features of tumor cells in terms of inflammation, metabolism, and exosomes and further investigate the potential mechanisms that underlie the dynamic communication between CAAs and BC cells, especially in obesity, which may result in neoteric therapeutic strategies. Addressing the clinical obstacles associated with obesity and metabolic syndrome will become increasingly important.

## CAAs secrete inflammatory factors that modify the behavior of breast cancer cells

**Fig. 1:**
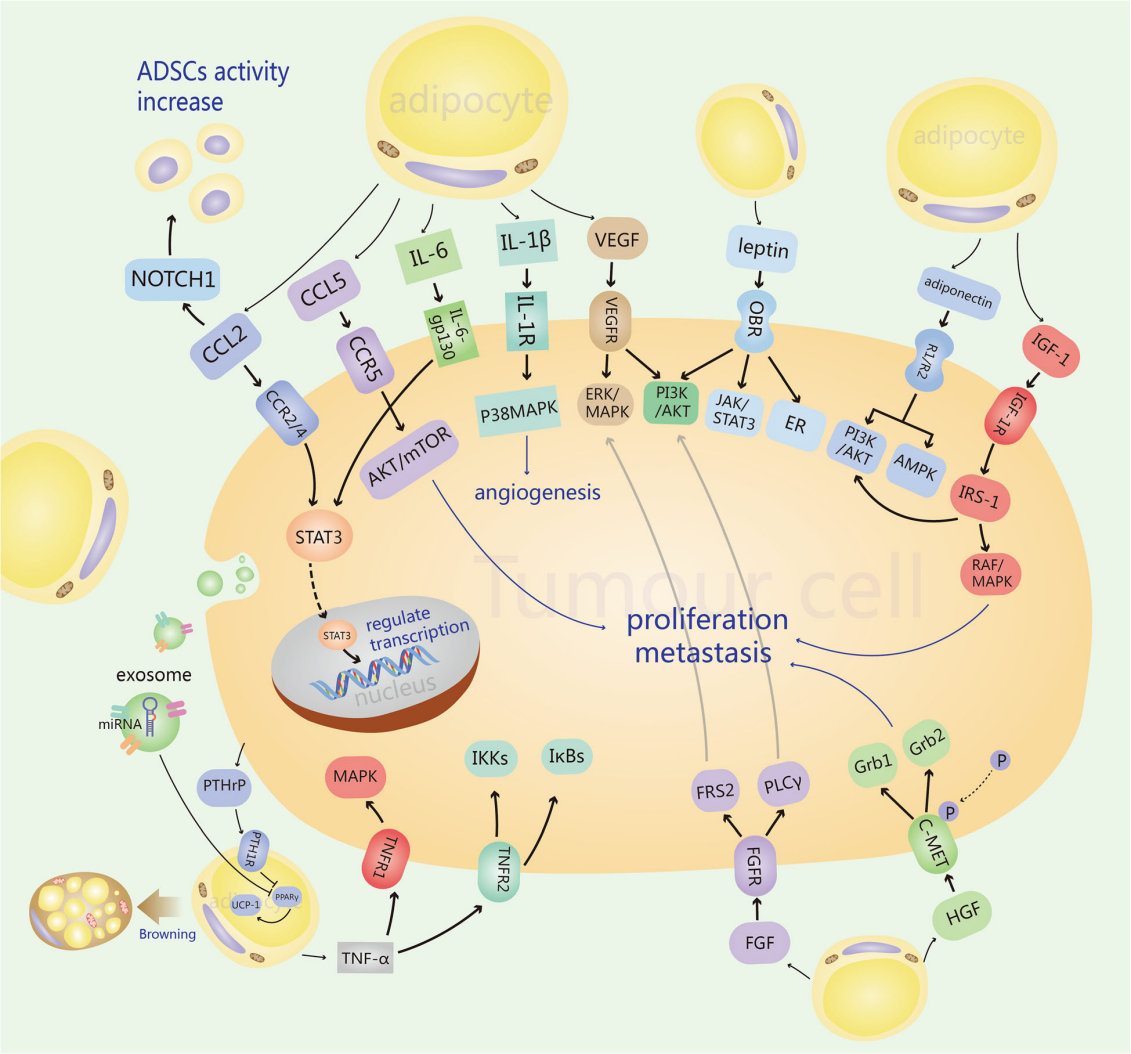
CAAs secrete inflammatory factors that modify the behavior of breast cancer cells

Breast adipocytes can be divided into three categories: mature adipocytes, preadipocytes, and adipose-derived stem cells (ADSCs). Limited studies have shown that there is a special type of adipocyte that exists in the surrounding matrix of invasive breast cancer [1]. Compared to normal adipocytes, this kind of adipocyte exhibits a series of characteristics, such as fibroblast-like phenotypes, smaller size, small and dispersed lipid droplets, overexpression of collagen VI, and low expression of adiponectin (APN) and other adipokines. This type of adipocyte is defined as cancer-associated adipocyte (CAA). CAAs secrete more chemokine (C–C motif) ligand 2 (CCL2) [6], chemokine (C–C motif) ligand 5 (CCL5) [7], interleukin-1β (IL-1β), interleukin-6 (IL-6) [1], tumor necrosis factor-alpha (TNF-α), vascular endothelial growth factor (VEGF), leptin [8], etc., which can promote the invasion and metastasis of breast cancer (Fig. 1).

### Chemokines

#### CCL2

Chemokine (C–C motif) ligand 2 (CCL2), also known as MCP-1 (monocyte chemoattractant protein-1), is located on chromosome 17q12, and the protein is composed of 76 amino acid residues. In the tumor microenvironment, CCL2 can be produced and secreted into the extracellular environment by many cells, such as cancer cells, fibroblasts, tumor-infiltrating monocytes, and endothelial cells. CCL2 works by binding to the G-protein-coupled receptor C–C motif chemokine receptors 2 and 4 (CCR2 and CCR4), and it is an effective inducible chemical factor for recruiting immune cells, especially monocytes, to the inflammatory region [9]. Santander et al. found that when E0771 breast tumor cells were co-cultured with macrophages and adipocytes, the expression of the chemokine CCL2 increased to recruit more adipocytes and monocytes/macrophages [10]. Tsuyada et al. found that breast cancer cells secrete cytokines that activate the signal transducer and activator of transcription 3 (STAT3) pathway in fibroblasts by activating the promoter of STAT3, which leads to an increase in the expression and secretion of CCL2. At the same time, in breast cancer cells, CCL2 can also induce the expression of NOTCH1 and the conduction of its downstream signals, thus inducing the activity of cancer stem cells (CSCs) [11]. In addition, the expression of CCL2 was significantly associated with neovascularization [12, 13]. Arendt et al. explored the mechanism of CCL2 in promoting angiogenesis. It was found that the expression of CCL2 and IL-1β was elevated in the adipose tissue associated with obesity and co-induced the secretion of chemokine (C–X–C motif) ligand 12 (CXCL12) in macrophages, which acted on blood vessels to enhance angiogenesis [14]. Their results also suggested that the mammary epithelial cells around the adipose tissue secreted CCL2, leading to the recruitment of macrophages and formation of the crown-like structures (CLS) associated with malignant progression of breast cancer. In conclusion, CCL2 mediates chemotaxis and angiogenesis by binding to CCR2 and CCR4.

#### CCL5

Chemokine (C–C motif) ligand 5 (CCL5, also known as RANTES) is located at chromosome 17q12, 8 kDa and plays an important role in many physiological processes. CCL5 can be produced by various cells, such as breast cancer cells and mesenchymal stem cells, and is highly expressed in breast cancer tissue [15]. D’Espositol’s study demonstrated that when MDA-MB-231 triple-negative breast cancer cells (TNBC) cells were co-cultured with human adipocytes, the level of CCL5 in the surrounding tissues increased, resulting in an enhanced invasion and metastasis ability of MDA-MB-231 cells [7]. Karnoub et al. discovered that breast cancer cells stimulate the secretion of CCL5 and that paracrine CCL5 reversibly binds to C–C motif chemokine receptor 5 (CCR5) on the membrane surface of MDA-MB-231 cells to enhance the migration, invasion, and metastasis of breast cancer cells [16]. Many studies have shown consistently that the CCL5-CCR5 axis is related to the invasion and metastasis of breast cancer [17–19]. Velasco-Velázquez’s study found that the CCL5-CCR5 axis is highly activated in TNBC− and human epidermal growth factor receptor-2 (HER2)-positive breast cancers and that CCR5+ cells respond to CCL5. When using the CCR5 inhibitors maraviroc and vicriviroc, the infiltration and invasion of breast cancer cells were reduced; this study also revealed that the invasion ability of CCR5+ cells isolated from breast cancer cells was 40 times greater than that of CCR5-cells [20]. Murooka et al. showed that CCL5 increases the proliferation and survival of MCF-7 cells overexpressing CCR5 in a mechanistic target of rapamycin (mTOR)-dependent manner [18]. Michael John Sax et al. explored the mechanism of the CCL5-CCR5 signaling pathway in promoting the metastasis of breast cancer. This study showed that inhibiting CCL5-CCR5 signaling in endothelial cells led to protein kinase B (AKT)/mTOR pathway activation defects as well as vascular and tumor growth defects in vitro and in vivo [19]. Therefore, it is likely that the stromal cells secrete CCL5 into the tumor microenvironment and that CCL5, by binding to CCR5, activates the AKT/mTOR pathway and promotes tumor metastasis. Furthermore, CCL5 partly impairs triglyceride synthesis in adipocytes through its cognate receptors via down-regulating lipogenic enzymes and sterol regulatory element-binding protein-1 (SREBP-1) [21]. CCL5 could also recruit macrophages [22], Th1, and Th17 effector T cells [23] into TME to induce and maintain inflammatory microenvironment. Consequently, CCL5 may be a potential target for precise treatment of breast cancer, but the specific mechanism needs to be investigated further.

### Inflammatory factors

#### IL-6 and IL-1β

Interleukin-6 (IL-6) is a cytokine that participates in a variety of biological activities, such as immune regulation, hematopoiesis, and tumorigenesis [24]. There are cancer-associated adipocytes (CAA) around breast cancer tissue. When adipocytes are co-cultured with breast cancer cells, the expression and secretion of IL-6 in adipocytes increase, promoting the invasion and migration of cancer cells [25–27]. When IL-6R is blocked by anti-IL-6R antibody, the metastasis of breast cancer induced by IL-6 is reversed [28, 29]. In addition, IL-6 promotes the proliferation and survival of tumor cells, as well as angiogenesis, by regulating Janus kinase (JAK)/STAT3 signaling pathways [30]. In HER2-positive breast cancer, IL-6 can also induce the production and maintenance of breast cancer stem cells (CSCs) through nuclear factor kappa-B (NF-κB) and STAT3 signaling pathways, then promoting tumor progression [31]. When Notch, Wnt and transforming growth factor-β (TGF-β) signaling pathways are activated, IL-6 also regulates self-renewal of breast cancer CSCs and promotes the survival and proliferation of CSCs [32]. Therefore, IL-6 promotes the invasion, metastasis, and angiogenesis of breast cancer mainly by activating JAK/STAT3 signaling pathway.

Blood monocytes, tissue macrophages, and other cells generate and release interleukin-1β (IL-1β) into the extracellular environment, which has pro-inflammatory effects and participates in the proliferation, invasion, metastasis, and angiogenesis of breast cancer [33, 34]. Moreover, adipocytes cultivated with cancer cells show an altered phenotype that the expression of pro-inflammatory factors is increased, including IL-1β [1]. IL-1β has been identified as a potential predictor of bone metastasis in breast cancer patients. IL-1β increases the expression of osteoprotegerin (OPG, a promoter of invasion and metastasis of breast cancer) and induces its secretion by activating the p38 mitogen-activated protein kinase (MAPK) signaling pathway [35]. Upon binding to IL-1R1, IL-1β increases the expression of VEGF and its receptors on endothelial cells (ECs), while activation of p38-MAPK signaling results in the migration of ECs and tube formation, both contributing to tumor angiogenesis [35].

#### TNF-α

Tumor necrosis factor-alpha (TNF-α) is an important inflammatory factor in the tumor microenvironment that is generated by tumor cells and stromal cells. The serum TNF-α level is significantly elevated in breast cancer patients. Moreover, the gene expression level of TNF-α showed a 5-fold increase in co-cultivated adipocytes [1], and the secretion of TNF-α was upregulated too [36]. TNF-α is considered as a potential biomarker of breast cancer growth and prognosis. TNF-α participates in multiple cellular signaling pathways by binding to receptors, which correlates with inflammation and survival of breast cancer [37]. Hagemann et al. confirmed that, in epithelial tumors, TNF-α stimulates the secretion of matrix metalloproteinases (MMP), thereby promoting the invasion of tumor cells [38]. Chua et al. showed that TNF-α enhanced the epithelial-mesenchymal transition of mammary epithelial cells, thus promoting the invasion and metastasis of breast cancer cells [39]. Another study also found that TNF-α induced the expression of VEGF to promote angiogenesis [40]. In addition, TNF-α activates transcription factors and related genes and then stimulates the related cell signaling pathways, which promotes the proliferation of tumor cells [41]. Therefore, TNF-α plays an important role in tumor proliferation, angiogenesis, invasion, and metastasis [42].

#### VEGF

Vascular endothelial growth factor (VEGF), consisting of two 23 kD single-chain proteins, has six isoforms and is widely expressed in tumor tissues. VEGF is highly upregulated in the breast tumor microenvironment, and VEGF/VEGFR activates oncogenic signaling pathways, including the MAPK pathway and the phosphatidylinositol 3-kinase (PI3K)/AKT pathway, triggering the proliferation, survival, migration, and angiogenesis of breast cancer cells [43]. Overexpression of VEGF-A in adipocytes induces angiogenesis and the rapid appearance of brown adipocytes in white adipose tissue. VEGF-A-induced browning is not associated with IL-4, contrary to traditional cold-induced browning [44]. Moreover, when VEGF was knocked down, MDA-MB-231 cells showed shorter perimeters and more rounded morphologies. VEGF also stimulates the migration of breast cancer by forming filopodia via the neuropilin-1 (NRP1)/ARHGAP17/Cdc42, signaling pathway [45]. Although anti-VEGF therapy has already been applied in clinical treatments, there is unavoidable resistance to this therapy eventually. A recent study demonstrated that high levels of serum IL-6 and fibroblast growth factor-2 (FGF-2) contributed to the resistance of anti-VEGF therapy in obese breast cancer patients and mouse models. However, this resistance could be overcome by blockade of IL-6 [46]. Thus, VEGF plays a crucial role in tumor angiogenesis and metastasis.

### Adipokines

#### Leptin and adiponectin

Leptin, by binding to the leptin receptor (OB-R), promotes breast cancer cells to proliferate and develop. The synthesis and plasma levels of leptin increase according to total adipose tissue mass, and leptin could also be produced by cancer-associated fibroblasts [47]. It has been reported that leptin levels are increased in the plasma of breast cancer patients, which correlates with higher grade, advanced tumor stages, and aggressive subtypes [36]. The generation and secretion of leptin were increased in cancer-associated adipocytes, compared to mature adipocytes [36]. Leptin activates estrogen receptor (ER) signaling, as well as the JAK/STAT3 and PI3K/AKT signaling pathways, promoting the proliferation of breast cancer cells [36]. Moreover, leptin increases the expression of cyclin D1 and cyclin-dependent kinase 2 [48], which accelerates the cell cycle of breast cancer cells. Leptin and IL-1 promote the expression of VEGF/VEGFR, in the meantime, leptin upregulates IL-1/IL-1R signaling both mRNA and protein levels, playing a pro-angiogenic role together [49]. Leptin could also promote proliferation in breast cancer cells in vitro via steroid receptor coactivator (SRC)-1/STAT3 signaling pathway [50].

The secretion of adiponectin decreases in CAA [36]. Adiponectin acts as a protective factor against tumor progression by binding to the adiponectin receptors AdipoR1 and AdipoR2. Adiponectin inhibits the growth and invasion of breast cancer cells and induces cell apoptosis by triggering Adenosine 5’-monophosphate (AMP)-activated protein kinase (AMPK) signaling and inhibiting PI3K/AKT signaling [36].

It seems that adiponectin acts in contrast to leptin, and the adiponectin to leptin ratio is used to describe this relationship. During the development of obesity, pre-adipocytes differentiate incorrectly, and the generation of hypoxia-inducible factor-1 (HIF-1) is induced by hypoxia to increase the expression of leptin and inhibit the expression of adiponectin. Therefore, the adiponectin to leptin ratio is decreased in obesity-related adipose tissue [51].

#### Parathyroid hormone-related protein

Parathyroid hormone-related protein (PTHrP) is processed into at least three different peptide products and generated by several kinds of cancer cells. PTHrP could activate a variety of signaling pathways in cells by binding to parathyroid hormone (PTH)/PTHrP receptor (PTH1R). In metabolic terms, PTHrP not only inhibits adipogenesis by downregulating peroxisome proliferator-activated receptor γ (PPARγ) expression [52] but also triggers adipose tissue browning by upregulating the expression of thermogenic genes such as uncoupling protein 1 (UCP1) [53]. Therefore, this suggests that tumor cell-derived PTHrP competes for limited nutrition to inhibit adipogenesis and obtains further energy from mature adipocytes by inducing lipolysis. Moreover, PTHrP could enhance bone metastasis, which may be closely related to bone marrow adipocytes [54]. Deleting PTH1R in the bone marrow adipose tissue contributes to the elevated expression of receptor activator of nuclear factor kappa B ligand (RANKL) and generates classic adipogenic markers which can induce mesenchymal stem cells to differentiate into mature adipocytes [55]. Therefore, it is speculated that tumor-originated PTHrP could result in cancer cachexia and promote bone metastasis by remodeling adipocytes.

## The metabolic reprogramming of CAAs drives cancer progression

**Fig. 2:**
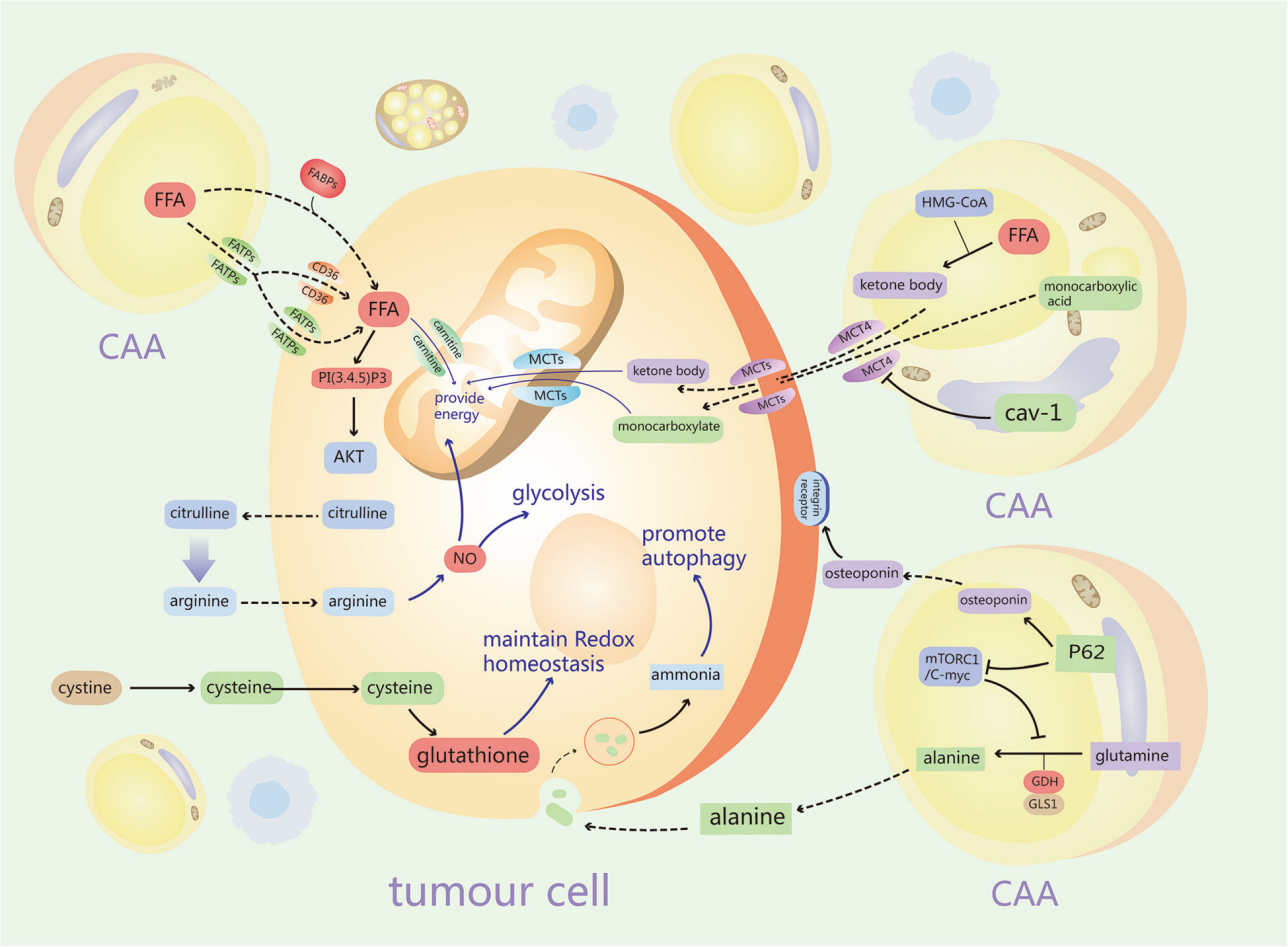
The metabolic reprogramming of CAAs promotes cancer progression

Upon interaction with breast cancer cells, adipocytes were initially identified as a tremendous energy storage that provides high-energy metabolites [10]. The metabolic reprogramming of CAAs can be attributed to their potential high tumor-promoting ability. It is speculated that tumors can induce reprogramming of metabolic synergy in adipocytes and adapt to intracellular metabolic processes to support proliferation through dynamic interactions between breast cancer cells and CAAs. In addition, the metabolic reprogramming that occurs in CAAs involves the metabolic regulation of almost all macronutrients, such as carbohydrates, lipids, and amino acids (Fig. 2).

### Glycolysis and monocarboxylate extrusion

To meet the extreme needs of energy of dividing cells, alterations happen in the metabolism of all macromolecules, such as proteins, carbohydrates, and lipids, in cancer cells [56]. As described in the “Warburg effect” [57], glycolysis increases in tumor cells despite the presence of oxygen [58]. Aerobic glycolysis provides the advantage of biosynthesis for tumor cells, and high-throughput substrates for glycolysis can efficiently divert carbon to key subsidiary biosynthetic pathways [56]. Furthermore, cancer has heterogeneity in the genetic and microenvironmental parameters that influence cell metabolism. The heterogeneity in tumor metabolism is not only demonstrated in the tumor itself, but also highlight the strong influence of the tumor microenvironment. The extent of tumor heterogeneity among different metabolic stages predicts metastatic specific organs. For example, cancer cells metastasized to the brain usually have flexible metabolic ability to utilize different fuels, such as acetate. And cancer cells metastasized to the liver could maximize the energy resources available to meet the energy demands [59]. However, previous studies on tumor metabolism have paid much attention to cancer cells themselves, ignoring different tumor “compartments” or mimicking of the tumor microenvironment. Recent studies have shown that in addition to cancer cells, different metabolic compartments, such as CAAs, in the tumor microenvironment play essential roles in the process [3, 60–62]. Glycolytic stromal cells promote tumor growth in breast cancer [63, 64]. The absence of Cav-1 is thought to be a hallmark of metabolic stress and is associated with poor survival in breast cancer [65]. Likewise, our results demonstrated that adipocytes co-cultivated with tumor cells exhibited increased UCP1 and monocarboxylate transporter 4 (MCT4) levels and reduced Cav-1 expression compared with single cultivation [66]. UCP1, which is generated only in beige/brown adipocytes, is also elevated in the adipose tissue close to breast cancer tissue [67]. Furthermore, increased expression of UCP1 leads to increased lipid mobilization and energy expenditure in vivo. Conversely, the activation of UCP1+ cells significantly decreases tumor development [67, 68]. In addition, monocarboxylate transporters (MCTs), which are transmembrane proteins, mediate the transport of diverse monocarboxylates, including lactate, ketones, and pyruvate and are related to poor prognosis in breast cancer. Of the MCTs, MCT1 and MCT2 are responsible for the uptake lactic acid to gain energy, while MCT4 facilitates lactic acid efflux to maintain a stable intracellular pH [69, 70]. Our results indicated that the overexpression of MCT1 is observed in hormone receptor-negative and high-proliferative subtypes and that when MCT4 is overexpressed in adjacent adipose tissue, the association between MCT1 expression and poor prognosis of breast cancer is further confirmed [71]. Moreover, it is clear that high expression of MCT2 in primary mammary-derived adipocytes (MGDAs) promotes the growth of breast cancer cells both in vitro and in vivo [72]. Meanwhile, MCT1 is a marker of mitochondrial metabolism (oxidative phosphorylation, OXPHOS), which is involved in the uptake of monocarboxylates and predominantly transports lactate into cells [69, 70]. Mitochondrial metabolism is more efficient and produces more adenosine triphosphate (ATP) to promote tumor proliferation and metastasis. In addition, the absence of caveolin 1 (Cav-1, caveolae protein) promotes aerobic glycolysis of stromal cells, as well as the secretion of energy-rich metabolites (for example, lactate and pyruvate), which are considered as energy resources for cancer cells and provide fuel for the mitochondria of adjacent cancer cells [73, 74]. Mechanistically, hypoxia induces the loss of Cav-1, which targets the MCT4 gene and upregulates its expression.

Overall, it is probably unsurprising that metabolic coupling has been formed between tumors and CAAs though the existence of a “monocarboxylate shuttle” in tumor-stromal interactions.

### Fatty acids and ketone bodies

Mammary fat consists of large lipid droplets, regulates lipid metabolism, and is a major component of energy storage. Cancer cells often invade the adipose tissue and induce adipocytes to release free fatty acids (FFA), which are absorbed by cancer cells and are used to produce ATP and facilitate tumor growth [3, 75, 76]. It is now widely recognized that FFAs may promote cancer progression through multiple mechanisms [77]. First, the sources of FFAs can act as structural units for newly synthesized membrane phospholipids and determine the phospholipid composition of membranes. Cancer cell membranes become enriched with saturated and/or mono-unsaturated fatty acids by enhancing de novo lipogenesis. In addition, due to the denser packaging of saturated lipids, increased lipogenesis also changes lateral and transverse membrane dynamics, resulting in cancer cells that are more resistant to oxidative stress-induced cell death and a limited uptake of drugs [77]. FFAs may also be used to supply energy for cells. Some specific types of tumors exhibit upregulated fatty acid uptake and elevated dependence on FFA β-oxidation as their main energy source [60–62, 78, 79]. Ultimately, FFAs can also be applied to the biosynthesis of a series of lipid-signaling molecules that promote tumorigenesis. For example, phosphatidylinositol-3,4,5-trisphosphate [PI(3,4,5)P3], a lipid messenger formed by the activation of PI3K that activates AKT to regulate cell proliferation and survival, plays a particularly important role in cancer progression [80]. Our studies showing that fatty acid transports such as CD36 and fatty acid transport protein-1 (FATP1) are primarily detected in most breast cancer tissues located adjacent to the adipose tissues support this idea; in contrast, the expression of fatty acid transports was elevated in adipocytes that were co-cultivated with breast cancer cells compared to the expression in adipocytes cultivated alone. Furthermore, adipocyte-induced CD36 and FATP1 expression contribute to cancer progression by fueling or signaling [61, 78, 81]. Fatty acid-binding proteins (FABPs) are also a series of proteins that serve as intracellular fatty acid transporters that regulate lipid trafficking and cellular responses [82]. The expression of FABP4 was found in ovarian cancer and acute myeloid leukemia at the adipocyte-tumor cell margin, providing fatty acids for rapid tumor growth [62, 79]. However, FABP4 expression is downregulated in the fat tissue surrounding breast cancer [1], while circulating, FABP4 levels in plasma are increased in breast cancer patients, and exogenous FABP4 accelerates breast cancer cell proliferation [83, 84]. Furthermore, FABP4 is also released from these macrophages, and the expression of fatty acid-binding proteins in tumor-associated macrophages (TAM) promotes breast cancer progression [82, 85], suggesting that macrophages, not adipocytes, may be the main source of FABP4 in patients with breast cancer. Moreover, our results indicated that FABP5 mRNA is highly expressed in adipocytes that were co-cultured with breast cancer cells [66]. FABP5 has also been shown to be overexpressed at the invasive front of the breast tumor and linked to poor survival [86, 87]. Similarly, Wang and colleagues described a novel mechanism in which FFAs were released by adipocytes after lipolysis induced by tumor secretions and were transferred into tumor cells to enhance FFA β-oxidation [3], in which FFA transformation maybe attributed to FABP5 high expression [86]. Thus, fatty acid metabolism patterns may alter based on the tissue-specific characteristics in cancers that are produced in the vicinity of adipose tissues and translated into the expected availability of these nutrients.

Ketone bodies are catabolites produced by fatty acid β-oxidation or aerobic glycolysis. Ketone bodies are produced and released by stromal cells and may upregulate cancer cell invasion by increasing OXPHOS [69, 88]. Moreover, it is well known that tumor cells need ketones as components of the tricarboxylic acid (TCA) cycle. Additionally, ketones are an ideal substrate for ATP production in invasive cancer cells because ketone bodies produce more ATP and consume less oxygen than glucose [89, 90]. For instance, breast cancer cells that were co-cultured with CAAs overexpress MCT2, inducing H3K9 acetylation, upregulating several tumor-promoting genes, and increasing the uptake of β-hydroxybutyrate to promote tumorigenesis [72]. CAAs are related to ketogenesis. The rate-limiting step in ketogenesis is the generation of 3-hydroxy-3-methyl glutaryl coenzyme A (HMG-CoA) by 3-hydroxybutyrate dehydrogenase 1 (BDH1), 3-hydroxymethyl-3-methylglutaryl-CoA lyase (HMGCL), and 3-hydroxy-3-methylglutaryl-CoA synthase 2 (HMGCS2) [88, 91]. Moreover, our results indicated that adipocytes co-cultivated with MDA-MB-231 cells exhibited increased activity of several rate-limiting enzymes, as exemplified by the strong increase in the mRNA levels of BDH1, HMGCL, and HMGCS2 [66]. Consistently, HMGCS2 was overexpressed in fibroblasts to accelerate breast cancer xenograft growth [88, 92]. In contrast, cancer cells exhibit high ketolytic activity and utilize ketone bodies by increasing acetyl-CoA acetyltransferase (ACAT) and 3-oxoacid CoA-transferase (OXCT) expression. Our results also showed that overexpression of ACAT 1/2 and OXCT 1/2 mRNA was observed in breast cancer cells in the presence of adipocytes. Similarly, high expression of OXCT or ACAT stimulates breast cancer growth and metastasis and is related to worse outcomes in breast cancer patients [88, 92, 93]. Furthermore, UCP1 expression has been observed to be increased in cancer-associated fibroblasts (CAFs) adjacent to breast cancer tissue, resulting in lipid mobilization and increasing ketone body production, which promotes tumor growth by supplying high-energy nutrients in a paracrine manner [94]. In addition, high expression of UCP1 was detected specifically in the resident adipose tissue and in adipocytes that were co-cultivated with tumor cells, demonstrating that CAAs may have a similar mechanism as that of CAFs.

In summary, FFAs and ketones play the role of paracrine high-energy nutrients in the “reverse Warburg effect” and can act as monocarboxylates to provide the energy required for cancer biomass synthesis, migration, and invasion.

### Amino acids

Amino acids, such as glutamine, glycine, serine, and proline, play important roles in cancer metabolic synergy [89, 95, 96]. In human breast cancer, asymmetric metabolism of these amino acids is also present between cancer and stromal cells.

Glutamine is an important amino acid for the mitochondrial metabolism of cancer cells and replenishes intermediate catabolites for the TCA cycle. Glutamine can be a pivotal source of the TCA cycle intermediates and ATP in cancer cells [97, 98]. Glutamine is also the substrate of the antioxidant glutathione and contributed to nucleotide synthesis [98, 99]. Recently, it has been recognized that stromal cells generate glutamine to promote cancer growth [100]. More recent studies on amino acids in the tumor microenvironment have demonstrated that glutamine can be obtained by cancer cells from the tumor microenvironment by increasing the activity of glutamate ammonia ligase, a key enzyme in glutamine synthesis [101]. Increased glutamine exposure induces the upregulation of glutamine importers and mitochondrial biogenesis in cancer cells, which results in resistance to cancer therapy [100, 101]. Likewise, glutamine could be provided by cultured astrocytes to brain cancer cells [102]. Furthermore, the secretion of alanine and ammonia accompanies glutamine catabolism. Stroma-associated pancreatic stellate cells could release alanine by relying on autophagy, and then cancer cells take up alanine as an alternative carbon source [103]. Ammonia facilitates autophagy and promotes autolysosomes to diffuse [104]. Cancer cells usually exhibit higher expression levels of glutamine metabolism enzymes such as glutaminase-1 (GLS1) and glutamate dehydrogenase (GDH) than those of stromal cells [105]. Mechanistically, several tumor stromal p62 levels decreased, which resulted in decreased consumption of glutamine through the mTORC1/c-MYC signaling pathway. Similarly, p62 deletion in adipocytes increased tumorigenesis through osteopontin secretion, and inhibition of mTORC1 caused adipocytes to regularly shut down energy utilization, which increased nutrient availability for cancer cells [106].

Adipose stromal cells in the tumor microenvironment have also been shown to metabolize symbiotically with cancer cells through arginine metabolis [107]. Citrulline and nitric oxide are generated by cancer cells by consuming and catabolizing arginine, which is mediated by inducible nitric oxide synthase. Nitric oxide not only suppresses oxidative phosphorylation but also facilitates glycolytic activity and proliferation. The citrulline produced in cancer cells is secreted into the extracellular matrix, taken up by stromal adipocytes, converted back into arginine, and released for cancer cells, thus forming this symbiotic metabolic shuttle. Cysteine, as one of the three amino acids of glutathione, contributes greatly to maintaining the redox balance. Given that cancer cells are often subjected to intense redox stress, especially when exposed to chemotherapies, the need for cysteine increases [108]. In blood, cysteine is mainly found in the form of oxidized dimers. Accordingly, cystine is consumed in cancer-associated bone marrow stromal cells to generate cysteine, which is released and then transported into the chronic lymphoid leukemia cells via neutral amino acid transporters. This cysteine in leukemia cells is used to generate glutathione and protect cancer cells from chemotherapy-induced cell death. Similarly, it was reported that ovarian CAFs secreted cysteine and glutathione to adjacent ovarian cancer cells, promoting chemoresistance [109]. In addition, it is reasonable to propose that the release of amino acids mediated by adipocytes may be a pathogenic factor related to worse survival outcomes.

## Exosomes interconnect cancer and cancer-associated adipocytes to promote tumor progression

Exosomes, a kind of small extracellular vesicle (30–100 nm), are derived from the endosomal compartments of almost all cells [110]. Moreover, exosome contents consist of mRNA, ncRNA, transcription factors, proteins, and lipids, and exosomes have been implicated in cell communication and the modulation of cell biology by trafficking these materials into target recipient cells [111]. In addition, exosomal miRNA profiles parallel those of the originating tumor cells [112]. It is clear that miRNA dysregulation plays a primary effect on tumor initiation, progression, and metastasis [113]. Although exosomes secreted from tumor cells are associated with tumor proliferation and metastasis [114], their potential effects on the neoplastic transformation of adipocytes remain unknown. Thus, we have demonstrated that the underlying mechanism involves the delivery of special onco-miRNAs such as miRNA-144, miRNA-126, and miRNA-155 from breast cells to adipocytes in the breast cancer microenvironment via exosomes, resulting in the conversion of resident adipocytes to CAAs [66]. Our study revealed that exosomal miRNA-144, acting as an important communicator between tumor cells and adipocytes, promoted the beige/brown differentiation of adipocytes, and exosomal miRNA-126 also played a vital role in the metabolic reprogramming of adipocytes [66]. In addition, exosomal miRNA-155 promotes lipolysis in adipocytes and facilitates an aggressive phenotype of tumor cells [115]. Moreover, exosomal miRNA-122 was highly expressed but has not been extensively studied, and exosomal miRNA-122 suppresses the uptake of glucose in premetastatic niche cells by decreasing the glycolytic enzyme pyruvate kinase, thereby facilitating disease progression [116]. Likewise, the expression of miRNA-105 secreted by breast cancer activates MYC signaling in CAFs and CAAs to induce a metabolic program. Furthermore, miR-105-reprogrammed CAFs enhance the metabolism of glucose and glutamine, fueling neighboring cancer cells in sufficient nutrient conditions, while these CAFs convert metabolic wastes such as lactic acid and ammonium into energy-rich metabolites in situations of nutrient deficiency. Therefore, the metabolic reprogramming of stromal cells mediated by miR-105 promotes sustained tumor growth by modulating the shared metabolic environment [117]. Similarly, HepG2, a hepatocellular carcinoma cell line, secreted exosomes, including some special proteins, activated phosphokinases and the NF-κB signaling pathway of adipocytes to promote tumor growth and angiogenesis and recruited more macrophages [118]. Moreover, this indicated that exosomal adrenomedullin originating from pancreatic cancer cells induces lipolysis by phosphorylating hormone-sensitive lipase in subcutaneous adipose tissue, which may be involved in the formation of CAAs [119]. As to circular RNA, exosomes released from gastric cancer cells deliver ciRS-133 (circular RNA sponge for miRNA-133) to pre-adipocytes, modulating the differentiation of pre-adipocytes to brown-like adipocytes through activating PR domain containing 16 (PRDM16) and inhibiting miRNA-133, which upregulates the expression of UCP1 [120]. In terms of exosome-released long noncoding RNAs, high expression of HOTAIR is found in multiple tumor tissues [121, 122]. Interestingly, cancer cells transport HOTAIR via exosomes to endothelial cells to stimulate angiogenesis by increasing VEGFA expression [123]. Although it has not been extensively shown that the effect of cancer-secreted long noncoding RNAs occurs in adipocytes, it should be further explored in future research. Taken together, these results show that exosome-derived contents are probably exploited by cancer cells as a sort of “signal” to convert the cells in the microenvironment into a pro-tumor niche.

Many studies have concentrated on exosomes derived from cancer cells that promote tumor proliferation and metastasis [114], while there is little work focused on exosomes from CAAs. It is evident that adipocyte exosomes are specifically enriched in proteins involved in fatty acid oxidation (FAO) and induce tumor cell metabolism reprogramming, which is beneficial for FAO stimulation of melanoma cell migration and invasion [124]. In conditions of obesity, the increased number of exosomes secreted and their effect on tumor aggressiveness contributed to the amplification of the harmful symbiosis between adipocytes and cancer cells [124]. In addition, the protein level of MMP3 in lung tumor tissues of obese patients is higher than that of nonobese patients. Mechanistically, adipocyte-derived exosomes increase MMP3, and MMP3 is transferred to lung cancer cells. MMP3 increases the activity of MMP9 in lung cancer cells and facilitates the invasion of cells in vitro and in vivo [125]. In terms of dysfunctional exosomal miRNAs, exosomal miR-21 expression was predominantly upregulated and potentially prognostic in multiplicate tumor stroma, such as triple-negative breast cancer and colorectal cancer [126, 127]. Equally higher levels of miR-21 in CAAs were observed, and miR-21 is transported from CAAs to cancer cells, inhibiting the apoptosis of ovarian cancer cells and producing chemotherapy resistance by combining with its direct new target apoptotic protease activating factor-1 (APAF1) [128]. miR-210, induced by HIF-1a, is overexpressed in tumor cells and in the tumor microenvironment and shows an inverse correlation with survival in patients with breast cancer [129, 130]. Meanwhile, miR-210 released from adipose-derived stem cells promoted the proliferation, invasion, and migration of endothelial cells by targeting runt-related transcription factor 3 (RUNX3), suggesting that exosomal miR-210 may mediate tumor angiogenesis [131]. Therefore, the exosome contents released by CAAs are tumor-supportive factors that induce drug resistance.

In summary, exosomes serve as a novel method of transmitting information among cells and play a pivotal role in the symbiosis between adipocytes and multiple types of cancer cells by regulating angiogenesis, immunity, and metabolism (Table 1).

**Table 1:** Exosomes emerge as an intercellular shuttle between cancer cells and CAAs

miRNA	Tumor/cell	Mechanism	References
miRNA-144	Breast cancer	miR-144 from cancer cells is able to target the MAP3K8 gene and reduce the phosphorylation level of ERK1/2, which leads to a decrease in the phosphorylation level of PPARγ S273 in adipocytes, ultimately leading to an increase in the expression of UCP1.	[66]
miRNA-126	Breast cancer	miR-126, derived from breast cancer cells, can target the IRS-1 gene to downregulate the expression of glut4 in adipocytes, which leads to a decrease in glucose uptake of adipocytes. And then AMPK is activated and protein levels of HIF-1α and MCT4 are increased, resulting in an increase of glycolysis and the secretion of metabolites, such as lactic acid and pyruvic acid.	[66]
miRNA-155	Breast cancer	miR-155 promotes beige/brown differentiation and remodels metabolism in adipocytes by downregulating the PPARγ expression.	[114]
miRNA-122	Breast cancer	miR-122 suppresses the uptake of glucose in premetastatic niche cells by decreasing the glycolytic enzyme pyruvate kinase, thereby facilitating disease progression.	[115]
miRNA-105	Breast cancer Hepatocellular carcinoma Pancreatic cancer	miR-105 activates MYC signaling in CAFs and CAAs to induce a metabolic program. And miR-105-reprogrammed CAFs enhance the metabolism of glucose and glutamine, fueling neighboring cancer cells in sufficient nutrient conditions, while these CAFs convert metabolic wastes such as lactic acid and ammonium into energy-rich metabolites in situations of nutrient deficiency.	[116–118]
ciRS-133	Gastric cancer	Exosomes released from gastric cancer cells deliver ciRS-133 to pre-adipocytes, modulating the differentiation of pre-adipocytes to brown-like adipocytes through activating PRDM16 and inhibiting miRNA-133.	[119]
miRNA-21	Breast cancer Colorectal cancer Ovarian cancer	miR-21 is transported from CAAs to cancer cells, inhibiting the apoptosis of ovarian cancer cells and producing chemotherapy resistance by combining with its direct new target APAF1.	[125–127]
miRNA-210	Endothelial cell	miR-210 released from adipose-derived stem cells promoted the proliferation, invasion and migration of endothelial cells by targeting RUNX3, suggesting that exosomal miR-210 may mediate tumor angiogenesis.	[130]

## Adipocytes play an important role in cancer

### Adipocytes are mediators between obesity and cancer

Adipocytes are the main matrix cell type in breast tissue. Because adipocytes surround breast cancer cells closely, the role of adipocytes in the evolution of breast cancer has recently been the focus of more attention. Most of the theoretical basis for studying the role of adipocytes in facilitating breast cancer evolution is associated with epidemiological evidence, suggesting a lower survival rate in obese breast cancer patients [3]. A key point is that we cannot identify whether the influence of adipocytes on breast cancer in obese individuals is different from that in normal or lean individuals based on the significant differences in adipocyte biology between thin and obese individuals, such as secretory protein spectrum changes, adipocyte size, and insulin sensitivity [4]. A laboratory study found that, compared with normal or thin women, the proliferation ability of MCF-7 cells increased when cultured with the conditioned media of adipocytes from obese women (body mass index (BMI): 30–35 kg/m^2^) [5]. Another study revealed that the estrogen-dependent cell E0771 in the mammary fat pad of mice fed a high-fat diet (a dietary-induced obesity (DIO) and hyperinsulinemia model) had a larger allograft volume than animals fed a normal diet [6]. These two studies showed the specific effects of obesity on breast cancer cell proliferation, whereas few studies have focused on exploring the mechanism by which the obese environment affects the progression of breast cancer. Although adipocytes may be the mediator between obesity and breast cancer, the specific mechanism remains to be elucidated.

### Adipocytes are a pivotal immunomodulatory factor in cancer

Moreover, the expression of programmed death-ligand 1 (PD-L1) in adipocytes prevents the anti-tumor function of CD8+ T cells. The lipogenesis inhibitor then selectively reduces PD-L1 expression in the adipose tissue and enhances the anti-tumor efficacy against PD-L1 or anti-PD-1.

Adipocytes contribute to immunosuppressive effects to avoid tumor elimination by the host antitumor immunity. Interestingly, the expression of PD-L1 has been shown to be significantly elevated in mature adipocytes compared to the level in preadipocytes. The expression of PD-L1 in adipocytes prevents the anti-tumor functions of CD8+ T cells. Then, the adipogenesis inhibitor selectively decreases the expression of PD-L1 in the adipose tissue but enhances the antitumor efficacy of anti-PD-L1 or anti-PD-1 [132]. Moreover, UCP-1 was overexpressed in cancer-associated fibroblasts resulting in the release of ATP-rich vesicles by activating autophagy [94], and our study verified that UCP-1 was also highly expressed in cancer-associated adipocytes after the uptake of tumor exosomes [115]. In obese models, ATP promotes a Th17 polarizing microenvironment by activating the P2X7 receptor pathway and increases the levels of IL-1β, IL-6, and IL-17 [133]. Moreover, IL-1β and IL-6 are also elevated in the tumor-adipocyte microenvironment [1] and interact with adipocytokines to aggravate tumor metastasis [134]. Meanwhile, crown-like structures (CLS) are characterized by adipocytes around infiltrating macrophages, and the CLS of the breast is found in nearly 50% of patient samples and is associated with lower survival [135]. ATP also increased the synthesis and release of CCL2 through activated P2Y2 receptors and attracted macrophages to the surrounding adipocytes [136]. Taken together, these findings imply that tumors may induce the release of ATP from stromal adipocytes to regulate the immune microenvironment and favor cancer progression.

### Potential drugs targeting CAAs

Inflammatory factors (CCL5, IL-6, etc.) and transporters (MCT1, CD36, etc.) are potential targets for breast cancer treatment. So far, there have been some targeted treatments used in the clinical practice or for clinical and preclinical studies, such as Cenicriviroc (inhibitor of CCR2) inhibiting CCL2 [137, 138], Maraviroc (inhibitor of CCR5) inhibiting CCL5 [139], Tocilizumab (monoclonal antibody of IL-6R) [140], Canakinumab (monoclonal antibody of IL-1β) [141], Infliximab (monoclonal antibody of TNF-α) [142–144], Bevacizumab (monoclonal antibody of VEGF-A) [145], AZD3695 (inhibitor of MCT1) [146], Nivolumab (monoclonal antibody of PD-1) [147], and Durvalumab and Avelumab (monoclonal antibodies of PD-L1) [148, 149] (Table 2). There are still many targets, such as inhibitor of exosomes, worth to be investigated for breast cancer precise therapy.

**Table 2:** Potential targets for breast cancer precise therapy

Agent	Target	In vitro effect	Preclinical and clinical effects	References
Cenicriviroc	CCR2	Inhibitor of CCR2	Inhibition of monocyte recruitment, Prevent virus from entering into a human cell	[137, 138]
Maraviroc	CCR5	Inhibitor of CCR5	Blockade the HIV from entering macrophages and T cells	[139]
Tocilizumab	IL-6R	Monoclonal antibody against IL-6R	Hindering IL-6 from exerting its pro-inflammatory effects.	[140]
Canakinumab	IL-1β	Monoclonal antibody against IL-1β	Treatment of cryopyrin-associated periodic syndromes	[141]
Infliximab	TNF-α	Monoclonal antibody against TNF-α	Treatment of autoimmune diseases	[142–144]
Bevacizumab (Avastin)	VEGF	Monoclonal antibody against VEGF-A	Angiogenesis inhibitor	[145]
AZD3695	MCT1	MCT1 inhibition	Reduce tumor growth, Increase intra-tumor lactate	[146]
Nivolumab	PD-1	Monoclonal antibody against PD-1	Checkpoint inhibitor	[147]
Durvalumab	PD-L1	Monoclonal antibody against PD-L1	Blocking the interaction of PD-L1 with the PD-1 and CD80 (B7.1) molecules.	[148]
Avelumab	PD-L1	Monoclonal antibody against PD-L1	Blocking the interaction of PD-L1 binding to PD-1	[149]

## Conclusion

In the microenvironment of breast cancer, the expression and secretory spectrum of inflammatory mediators of adipocytes are changed, and the secretion of chemokines CCL5 and CCL2 and the inflammatory factors IL-6 and TNF-α are increased, which further promotes the proliferation and invasion of tumor cells and the formation of neovascularization. It has been shown that the inflammatory microenvironment of breast cancer is an important driving factor for the progression of the disease and may become a potential target for new treatments. The current research has demonstrated that, especially in obese individuals, tumor cells and adipocytes have a clear clinical relevance, which helps to deepen our understanding of the mechanism of breast cancer. New drugs can be developed to target adipocytes and/or cancer cells directly or can be used in adjuvant therapy to achieve the maximum effect of existing treatment. Because obesity can affect the incidence and progression of cancer, it may weaken the therapeutic effect of cancer treatments. For obesity, direct lifestyle intervention through diet and exercise may be the simplest and most effective strategy.

We believe that an in-depth understanding of exosomes in the tumor microenvironment may contribute to the design of cancer-diagnostic and cancer-prognostic tools. Effective therapeutic strategies for cancer using exosomes as drug carriers are expected in the near future.

## Data Availability

Not applicable.

## Abbreviations

ACAT: Acetyl-CoA acetyltransferase
ADSCs: Adipose-derived stem cells
AKT: Protein kinase B
AMPK: Adenosine 5’-monophosphate (AMP)-activated protein kinase
APAF1: Apoptotic protease activating factor-1
APN: Adiponectin
ATP: Adenosine triphosphate
BC: Breast cancer
BDH1: 3-Hydroxybutyrate dehydrogenase 1
BMI: Body mass index
CAAs: Cancer-associated adipocytes
CAFs: Cancer-associated fibroblasts
Cav-1: Caveolin 1
CCL2: Chemokine (C–C motif) ligand 2
CCL5: Chemokine (C–C motif) ligand 5
CCR2,4,5: C–C motif chemokine receptor2,4,5
CLS: Crown-like structures
CSCs: Cancer stem cells
CXCL12: Chemokine (C–X–C motif) ligand 12
DIO: Dietary-induced obesity
ECs: Endothelial cells
ER: Estrogen receptor
FABPs: Fatty acid-binding proteins
FAO: Fatty acid oxidation
FATP1: Fatty acid transport protein-1
FFAs: Free fatty acids
FGF-2: Fibroblast growth factor-2
GDH: Glutamate dehydrogenase
GLS1: Glutaminase-1
gp130: Glycoprotein 130
HER2: Human epidermal growth factor receptor-2
HIF-1: Hypoxia-inducible factor-1
HMGCL: 3-Hydroxymethyl-3-methylglutaryl-CoA lyase
HMG-CoA: 3-Hydroxy-3-methyl glutaryl coenzyme A
HMGCS2: 3-Hydroxy-3-methylglutaryl-CoA synthase 2
IL-1β: Interleukin-1β
IL-6: Interleukin-6
JAK: Janus kinase
MAPK: Mitogen-activated protein kinase
MCP-1: Monocyte chemoattractant protein-1
MCT4: Monocarboxylate transporter 4
MCTs: Monocarboxylate transporters
MGDAs: Mammary-derived adipocytes
MMP: Matrix metalloproteinases
mTOR: Mechanistic target of rapamycin
NF-κB: Nuclear factor kappa-B
NRP1: Neuropilin-1
OB-R: Leptin receptor
OPG: Osteoprotegerin
OXCT: 3-Oxoacid CoA-transferase
OXPHOS: Oxidative phosphorylation
PD-L1: Programmed death-ligand 1
PI(3,4,5)P3: Phosphatidylinositol-3,4,5-trisphosphate
PI3K: Phosphatidylinositol 3-kinase
PPARγ: Peroxisome proliferators-activated receptor γ
PRDM16: PR domain containing 16
PTHrP: Parathyroid hormone-related protein
RANKL: Receptor activator of nuclear factor kappa B ligand
RUNX3: Runt-related transcription factor 3
SRC: Steroid receptor coactivator
SREBP-1: Sterol regulatory element-binding protein-1
STAT3: Signal transducer and activator of transcription 3
TAM: Tumor-associated macrophages
TCA: Tricarboxylic acid
TGF-β: Transforming growth factor-β
TNF-α: Tumor necrosis factor-alpha
UCP1: Uncoupling protein 1
VEGF: Vascular endothelial growth factor
